# Anxious, depressed: Should medical schools screen their students?

**DOI:** 10.30476/jamp.2020.86182.1224

**Published:** 2021-07

**Authors:** DEEBAN RATNESWARAN, MUHAMMED KERMALI, TECK K KHONG

**Affiliations:** 1 Lane Fox Unit, Sleep Disorders Centre, Guy’s and St Thomas’ , HS Foundation Trust, London, United Kingdom; 2 Department of Asthma, Allergy and Lung Biology, King’s College London, London, United Kingdom; 3 Institute of Medical & Biomedical Education, St George’s, University of London, London, United Kingdom

## Dear editor

It is generally recognized that medical students enter a demanding and competitive environment during their undergraduate studies and that this may cause significant stress ( 1 ). The latter may contribute to or even cause adverse effects including high levels of anxiety, depression and the use of drug and alcohol amongst medical students ( 2 , 3 ).

In an independent charitable course aimed at supporting 2^nd^ year medical students preparing for clinical examinations, the attendants were informally invited to complete questionnaires anonymously relating to their mental well-being. We were surprised to find significantly high levels of clinically ‘abnormal’ anxiety (43%; mean score 10.3±3.2) and depression (12%; mean score 8.9±2.4), as measured by the Hospital Anxiety and Depression Scale (HADS). Furthermore, the Centre of Epidemiological Studies Depression Scale – 10 (CESD-10) considered 34% of these students as depressed (mean score 8.9±4.7).

Despite wide reported differences ( 2 ), it seems likely that stress and anxiety levels peak as examinations approach ( 4 ). For some individuals, if other negative life events however small occurred simultaneously during this period, they may not have the cognitive resource to deal with this effectively and may succumb to major psychological adversity.

Medical schools *do* actively create support systems to help those suffering from adversity ( 5 ). However, this relies on the student being forthcoming. Fear of stigmatization, given the future role as a doctor is a real barrier to help seeking ( 6 ). A perceived lack of time, secondary to work-load, may also be an important factor in not seeking help, especially in a performance and deadline driven student cohort, used to excelling academically( 7 ). Students that feel isolated, who are introverted in personality, or do not have supportive social groups are particularly at risk ( 8).

Our informal survey prompted us to suggest a more radical approach whereby, at peak levels of stress, students are *screened* with validated anxiety and depression scores. This would work two-fold: firstly by encouraging the students to reflect on their own, perhaps ignored symptomatology; but secondly, by providing universities with information, and trends, to identify and approach students who might be at risk. Utmost sensitivity and confidentiality are paramount in making this successful, but we are unaware of any previous implementation of this technique. We urge its consideration when renewing school health policy.
